# Impact of Rapid Urbanization on the Rates of Infection by *Vibrio cholerae* O1 and Enterotoxigenic *Escherichia coli* in Dhaka, Bangladesh

**DOI:** 10.1371/journal.pntd.0000999

**Published:** 2011-04-05

**Authors:** Fahima Chowdhury, Mohammad Arif Rahman, Yasmin A. Begum, Ashraful I. Khan, Abu S. G. Faruque, Nirod Chandra Saha, Nabilah Ibnat Baby, M. A. Malek, Anisha Rajeev Kumar, Ann-Mari Svennerholm, Mark Pietroni, Alejandro Cravioto, Firdausi Qadri

**Affiliations:** 1 International Centre for Diarrheal Disease Research, Dhaka, Bangladesh; 2 Institute of Biomedicine, Department of Microbiology and Immunology, University of Gothenburg, Gothenburg, Sweden; University of Maryland, United States of America

## Abstract

**Background:**

In Bangladesh, increases in cholera epidemics are being documented with a greater incidence and severity. The aim of this prospective study was to identify the prevalence and importance of *V. cholerae* O1 and enterotoxigenic *Escherichia coli* (ETEC) as causal agents of severe diarrhea in a high diarrhea prone urban area in Dhaka city.

**Methodology:**

Systematic surveillance was carried out on all diarrheal patients admitted from Mirpur between March 2008 to February 2010 at the ICDDR, B hospital. Stool or rectal swabs were collected from every third diarrheal patient for microbiological evaluation.

**Principal Findings:**

Of diarrheal patients attending the hospital from Mirpur, 41% suffered from severe dehydration with 39% requiring intravenous rehydration therapy. More diarrheal patients were above five years of age (64%) than those below five years of age (36%). About 60% of the patients above five years of age had severe dehydration compared with only 9% of patients under five years of age. The most prevalent pathogen isolated was *Vibrio cholerae* O1 (23%) followed by ETEC (11%). About 8% of cholera infection was seen in infants with the youngest children being one month of age while in the case of ETEC the rate was 11%. Of the isolated ETEC strains, the enterotoxin type were almost equally distributed; ST accounted for 31% of strains; LT/ST for 38% and LT for 31%.

**Conclusion:**

*V. cholerae* O1 is the major bacterial pathogen and a cause of severe cholera disease in 23% of patients from Mirpur. This represents a socioeconomic group that best reflects the major areas of high cholera burden in the country. Vaccines that can target such high risk groups in the country and the region will hopefully be able to reduce the disease morbidity and the transmission of pathogens that impact the life and health of people.

## Author Summary

Bangladesh is a country where acute dehydrating diarrhea or cholera is common and is seen at least two times every year and additionally in natural disasters. In addition cholera cases have increased in the country, especially in urban settings such as in the capital city, Dhaka, where the number of hospitalized patients with more severe disease has tremendously increased. In the present observation, we have concentrated on determining the occurrence of diarrhoea caused by the two most common bacterial agents *V. cholerae* O1 and enterotoxigenic *Escherichia coli* (ETEC) in a densely populated, disease prone area Mirpur in Dhaka for two years from March 2008 to February 2010. Stool or rectal specimens from diarrheal patients coming to the ICDDR,B hospital from Mirpur were tested for the two bacterial pathogens. We found that *V. cholerae* O1 was the major bacterial pathogen and a cause of severe cholera disease in 23% of patients (2,647 of a total of 11,395 patients) from Mirpur. We surmise that cholera vaccines, as well as other public health tools that can target such high risk groups in the country, will be able to reduce the disease morbidity and the transmission of pathogens to improve the quality of life in urban settings.

## Introduction

Epidemics of acute watery diarrhea are on the increase in many countries around the world with major outbreaks being seen in Asia and Africa [1] in both rural and urban areas. In Bangladesh, such increases in diarrheal epidemics have been documented [2], [3], [4], [5] with a greater incidence in the capital city, Dhaka. Not only has the number of diarrheal patients seeking care increased but also the severity of disease [2], [4], [5]. The epidemics in Bangladesh have occurred during floods, cyclones and other natural disasters [6], as well as in the biannual seasonal periods [7]. *Vibrio cholerae* is the most frequently isolated bacterial pathogen from patients presenting with diarrhea to hospitals [2], [3], [5]. Comparing diarrheal epidemics in Dhaka from 2007, 2004, and 1998, more severe dehydration due to cholera was seen in 2007 (35%) compared with 2003 (25%) and 1998 (22%) [2]. During both the 1998 and 2004 flood associated epidemics; there was an approximate doubling in the proportion of patients with *V. cholerae* infection compared with the seasonally matched control period (1998; 42% from 20%; 2004 23% from 11%). A similar trend was observed in epidemics in rural Bangladesh, with over 70% of patients presenting with severe diarrhea in 2006, compared to 30–40% in the late 1990s [4]. The aim of this prospective study was to identify the prevalence and importance of *V. cholerae* O1 and *Escherichia coli* (ETEC) as causal agents of severe diarrhea in Dhaka. The objective was to focus on acute watery diarrhea which required hospitalization to obtain information that could serve as baseline data for carrying out vaccine related as well as water and sanitation based preventive measures in the near future. Our earlier retrospective data from the ICDDR,B have showed that of patients from about 13 areas of Dhaka city that seek help at the diarrheal hospital, the highest hospitalization is from the Mirpur area of Dhaka city [8].

We have in the present study carried out a systematic surveillance of patients attending the ICDDR, B diarrheal hospital in Dhaka city from Mirpur. This area is a densely populated area of a mixed income neighborhood with a population of about 2.5 million, representing other populated areas in the region. To better understand the etiology of diarrheal disease we carried out a demographic, clinical and microbiological outcome analyses. We placed emphasis on determining the prevalence of *V. cholerae* O1 induced acute watery diarrhea over the 24 month study period between 2008 to 2010. The prevalence of another major cause of watery diarrhea, ETEC which is a common pathogen in these setting was also studied [7], [9].

## Methods

### Study site

This study was carried out in patients attending the diarrheal hospital at the ICDDR, B, Dhaka, Bangladesh. Previous data analysis of hospitalized patients due to severe diarrhea showed the highest percentage coming from the Mirpur area of the city [8]. Systematic surveillance was carried out on all diarrheal patients admitted from Mirpur between March 2008 to February 2010. The metropolitan area of Dhaka has a total area of 153.84 Sq.Km, is divided into 10 zones. [10]. The Mirpur area is in the north-west of the city consists of two zones that are subdivided into 16 metropolitan wards.

### Ethics statement

The hospital surveillance activities were approved by the Research review committee and ethical review committee of ICDDR,B. Informed oral consent was obtained due to most of the participants were illiterate. According to the ICDDR,B hospital surveillance system, we only require verbal consent from patients undergoing routine investigation for collecting only stool specimens. Consent was documented in the surveillance questionnaire.

### Patient and clinical data

Demographic, socioeconomic, and clinical data for each patient was captured in the ICDDR,B database and clinical examination and history forms were completed by trained hospital physicians. All patients were assessed for clinical conditions, including degree of dehydration according to WHO guidelines [11] with treatment being given according to the ICDDR,B protocol [12]. The clinical criteria for admission were moderate to severe diarrhea requiring hospitalization. The WHO categorization for exclusive breast feeding practice in infants 6 months and younger was also applied [13], [14].

### Microbiological evaluation

Stool or rectal swab specimens were collected from every third diarrheal patient coming from Mirpur who were admitted to the ICDDR, B hospital and were evaluated for *V. cholerae* O1 and O139 and also tested for ETEC [9], [15]. In addition specimens were tested for *Salmonella* spp., and S*higella* spp. using standard microbiological techniques [16] Other diarrheagenic *E. coli* were not analyzed in the study. For isolation of *V. cholerae*, specimens were cultured on taurocholate-tellurite-gelatin agar plates. Specific monoclonal antibodies were used to detect *V. cholerae* O1, Ogawa and Inaba serotypes, as well as O1 or O139 serogroups [17], [18]. For microbiological evaluation of *V.cholerae*, specimens were also enriched in alkaline peptone water for 4 hours and then cultured [3]. The variant phenotype of cholera toxin expressed by *V. cholerae* O1 was detected by PCR [19]. For this purpose, a random collection of every 100^th^ strain of *V. cholerae* O1 in the collection (n = 57) were typed as the El Tor or the altered phenotype [20].

For ETEC detection, *E. coli* was cultured overnight on MacConkey agar plates and six freshly lactose-fermenting colonies were isolated and tested for the presence of heat labile toxin (LT) and heat stable toxin (ST) [9], [18]. Detection of LT was carried out using a ganglioside GM1 ELISA test [21] and ST was detected by an inhibition ELISA [22], [23]. Colonies that tested positive for either toxin were plated onto colonization factor antigen (CFA) agar with bile salts to identify the CFs using a dot blot immunoassay technique with specific monoclonal antibodies [9], [24]. Information concerning the prevalence of rotavirus was obtained for the study period from the available ICDDR, B database but was not prospectively determined for patients coming from the Mirpur area. Nutritional status of the children was expressed in terms of Standard Deviation Score (SD) of an anthropometric index such as weight for age, Height for age or weight for height. The anthropometric measurement for children's WAZ (“Weight-for-age z-score”), HAZ (“height-for-age z-score”) or WHZ (“Weight-for-Height z-score”.) was calculated in relation to the new World Health Organization growth standards [48]. We considered <−2 Z score as underweight, stunted and wasted respectively for the children.

### Statistical analysis

Statistical analyses were performed using Statistical Package for Social Sciences (SPSS, Chicago, IL) version 12.0. Associations were carried out by calculating the odds ratio (OR) with 95% confidence intervals (CI) using EpiInfo 3.3.2 and χ^2^ (chi-square) tests. Areas maps were prepared using Adobe Photoshop 7.0.

## Results

### Socioeconomic and demographic features

Of all patients admitted to the ICDDR,B hospital during the study period, 31,588 (12%) came from the Mirpur area. Diarrheal patients from all 16 wards of the area came to the ICDDR, B hospital for treatment. We however, identified 6/16 metropolitan wards within Mirpur which had a high rates of acute diarrhea based on the patient hospitalization information while the rest had moderate to lower rates. The median age was 18 years (1 mo-96 yr) with 54% male and 46% female patients (Table 1). In terms of the Mirpur patients, 89% lived in low income housing, and only 8% lived in independent homes or in high income residential areas. About 20% of adult males, either the patients or the parent's of admitted children, had received formal schooling, while for females this percentage was lower, falling, between 11–12%. Over 98% of patients used tap water and around 1% used tube-well water for bathing or drinking purposes. About 33% of patients did not use treated drinking water, and 65% reported that they boiled their water. The majority of the study population (92%) used shared sanitary latrines in the community. Of the diarrheal children aged up to 6 months of age, only 14% were exclusively breastfed. About 77% of the study population attending the hospital from Mirpur had a low monthly income (≤10,000 taka ∼US$ 150).

**Table 1:** Demographic and clinical characteristics of patients from Mirpur between March 2008 and February 2010.

Socio Demographic Features	Patients N = 31,588 (%)
Median age (years)	18
<1 years	3329 (10)
1 to 5 years	8088 (26)
6 to 17 years	3386 (11)
≥18 years	16785 (53)
Male	17114 (54)
Monthly income ≤10,000 taka	24330 (77)
Treatment of drinking water	21102 (67)
**Clinical Features**	
Fever (>37.7°C)	1100 (4)
Diarrhea for >4 days	767 (2)
Watery stool	30348 (96)
Bloody stool	469 (2)
Abdominal pain	18269 (58)
Vomiting prior to admission	25001(79)
Severe dehydration on presentation	12877 (41)
Use of ORS before arrival	28994 (92)
Use of I.V before arrival	1311(4)
Use of medication before arrival	16436(52)
Intravenous rehydration	12403 (39)
ORS used	13554 (43)

### Clinical characteristics of patients

Of the diarrheal patients attending the hospital from Mirpur, 41% suffered from severe dehydration and 39% were given intravenous rehydration therapy (Table 1). About 4% of the patients had fever (>37.7°C); 58% had abdominal pain; and 79% suffered from vomiting. More than half of the patients (52%) took antibiotics or had oral rehydration (92%) before they were hospitalized. There were more diarrheal patients above 5 years of age (64%; median: 27 yr) than those below five years of age (36%; median: 1 yr) who were admitted to the hospital from Mirpur. About 60% of the patients above 5 years of age had severe dehydration compared with only 9% of patients under 5 years of age.

Among the children under 5 years of age, 40% (N =  = 1717) were moderate to severely under weight, 52% were stunted (N = 2225) and 22% (N = 902) were wasted. One of the definitions of HAZ is “height-for-age z-score”, WAZ is “Weight-for-age z-score” and WHZ is “Weight-for-Height z-score”. Among cholera and non cholera pediatric patients WAZ<−2 Z score 43% vs. 39% (p = 0.03), HAZ<−2 Z score 53% vs. 50% (p = ns), WHZ<−2 Z score 27% vs. 19% (p = 0.001).

### Bacterial pathogens isolated from stool samples

**Figure 1:**
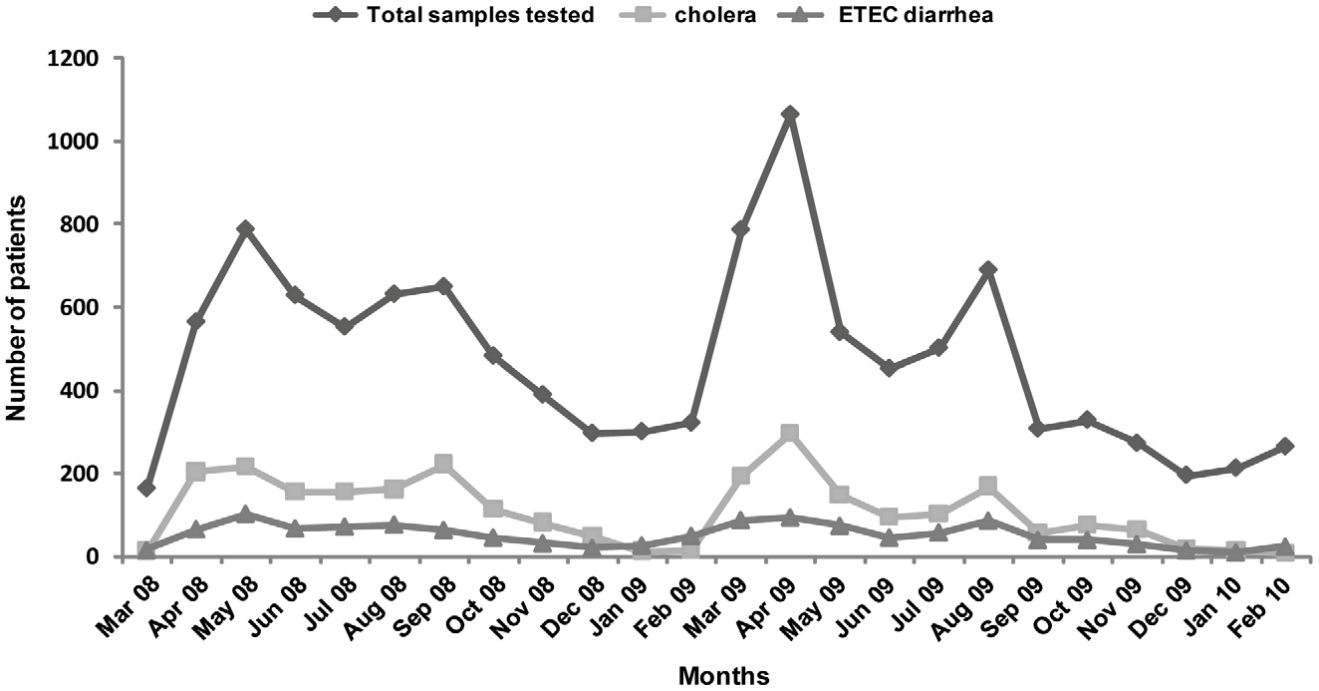
Seasonality of cholera and ETEC diarrheal patients.

The most prevalent pathogen isolated from diarrheal patients coming from Mirpur during the study period was *Vibrio cholerae* O1 (23%; n = 2647). This was followed by ETEC (11%; n = 1248). In addition, 230 patients (2%) were positive for both *V. cholerae* O1 and ETEC. Cholera rates showed two peaks, one between April and May and the second between August and September with lower rates in the winter months beginning in November. In terms of ETEC rates, the disease increased from April and continued until September with lower levels in the winter months (Figure 1). Less than 1% of the specimens were positive for *Salmonella spp* and *Shigella spp.* [20].

### Diarrheal hospitalization rates in Mirpur

**Figure 2:**
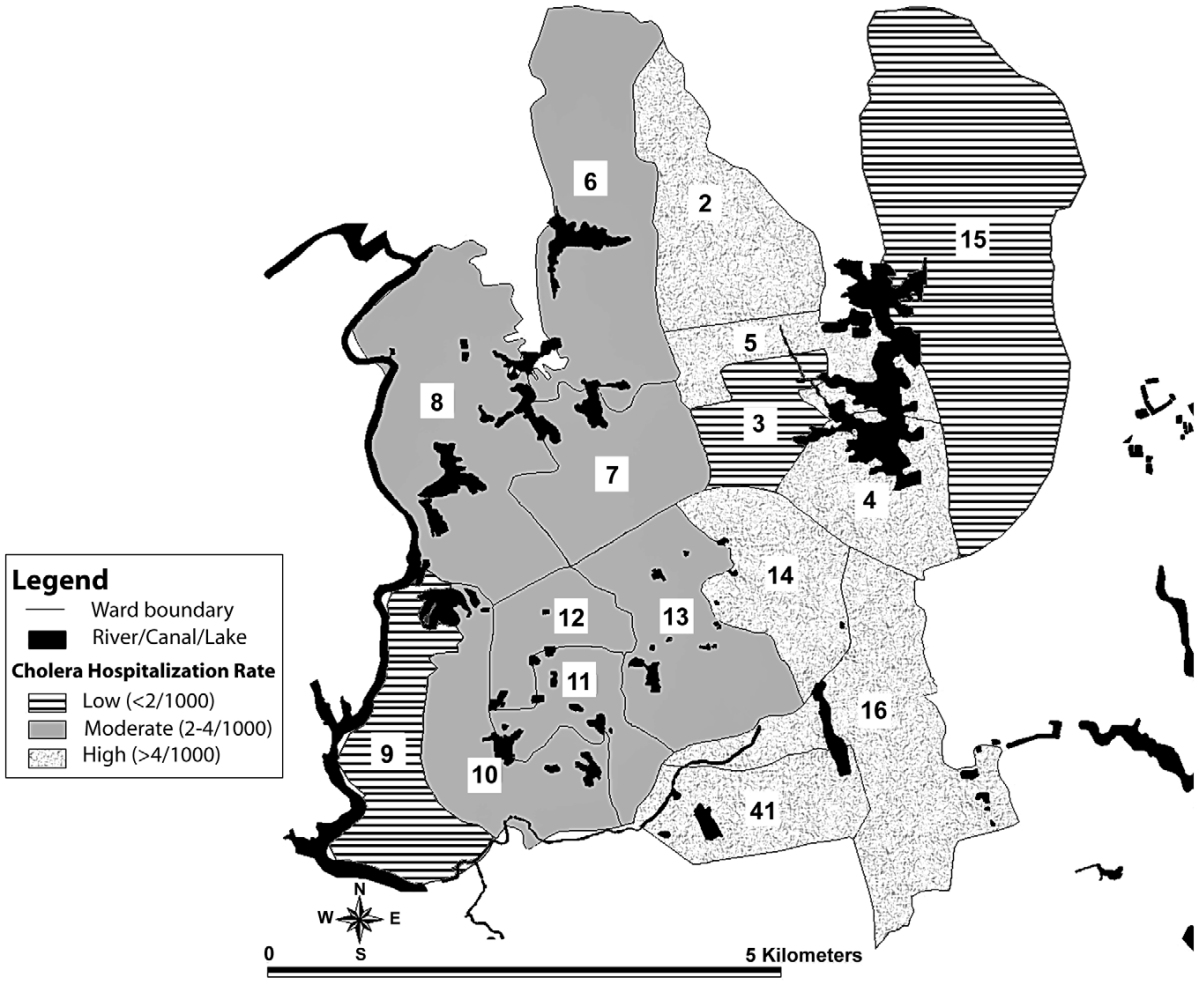
Cholera hospitalization rate (ICDDR, B, Dhaka hospital) from Mirpur area.

The Mirpur area comprises zones 7 and 8 of the city which include 16 metropolitan wards. Diarrheal hospitalization rates were highest from wards 2, 4, 5, 14, 16 and 41, moderate (>2 to 4/1000) from wards 6, 7, 8, 10, 11, 12 and 13, and lower (less than 2/1000) for the rest of wards (1, 3, 9 and 15) from where patients attended the hospital (Figure 2). ETEC diarrheal hospitalization rates were lower and ranged from 2.3–3.5/1000 population with wards 5, 11, 14, 16 and 41 showing higher rates. In terms of infection by both *V. cholerae* and ETEC, patients came from wards 5, 14, 16 and 41, although rates of cholera were higher in wards 2 and 4.

About 23% (n = 2647, median: 20 yr) of patients had culture-confirmed cholera. Severe dehydration was seen in 70% of cholera patients. About 77% were over five years of age (median: 23 yr). Among cholera patients, 80% of patients from Mirpur above 5 years of age suffered from severe dehydration, but children less than 5 years of age (median: 2 yr) this percentage was lower at 35%. About 8% of cholera infection was seen in infants (median: 3 mo) with the youngest children being only 1 month of age (n = 186). Of the *V. cholerae* O1 strains isolated from Mirpur patients, 82% were Ogawa and 18% were Inaba serotypes. Screening of a random number of *V. cholerae* O1 strains (around every 100^th^ strain isolated during the period; 42 strains in total), showed these were all variant strains that produced the classical phenotype *V. cholerae* O1 toxin.

In terms of overall ETEC rates among Mirpur patients, 11% had ETEC diarrhea ((N = 1248, median: 18 yr) with 38% suffering from severe dehydration. Of these, 59% were more than 5 years of age (median: 28 yr) while 41% were under 5 years of age (median: 1 yr). Of the under 5 year old in age, 10% suffered from severe dehydration where as 57% adults with ETEC diarrhea were severly dehydrated. Among children less than 5 years of age (median: 1 yr), 12% had ETEC diarrhea.

**Figure 3:**
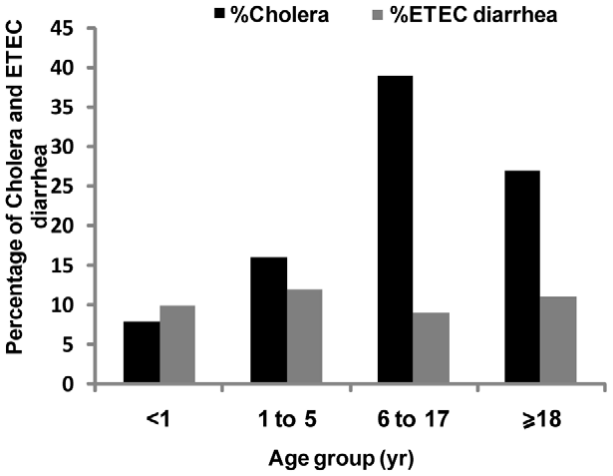
Cholera and ETEC hospitalization rate among the patients of different age group coming from Mirpur area.

The cholera hospitalization rate for children below 1 year of age was 8% while in the case of ETEC, it was 11% (Figure 3). All severely dehydrated patients received azithromycin in the hospital with an average duration of stay of 9 hours.

### Comparison of clinical features of cholera and ETEC diarrheal patients

We analyzed data to determine if there were any differences between disease presentation of patients admitted with cholera or ETEC diarrhea. There were more cholera patients than ETEC diarrheal patients admitted to the hospital (*P*<0.001). The highest numbers of cholera and ETEC diarrheal patients were above 18 years of age. The second highest age group of cholera patients was in the 1–17 year range, whereas ETEC diarrhea patients fell into the 1–5 year range (Table 2). Fever was more common in ETEC than *V. cholerae* O1 infection (4% versus 1%; *P* = 0.001). Cholera patients had significantly higher rates of watery stools than ETEC diarrheal patients (99% vs. 97%, respectively; *P* = 0.001) but no difference in abdominal pain was seen between the patient groups. Cholera patients showed higher rates of profuse vomiting at more than ten times prior to hospital admission when compared with ETEC patients translating into a higher risk for vomiting of 20% versus 11%, respectively (*P*<0.001). Severe dehydration was also more common in cholera than ETEC diarrheal patients (70% vs. 38%, P<0.001) as were the rates for intravenous fluid requirement (65% vs. 37%, P<0.001).

**Table 2:** Clinical features of cholera and ETEC diarrheal patients during study period from Mirpur.

	CholeraN = 2647 (%)	ETEC diarrheaN = 1248 (%)	Odds ratio(95% CI)	*P* value(χ^2^ test)
Median age (yr)	20	18		
<1 (yr)	101(4)	130 (11)	0.34 (0.26–0.45)	<0.001***
1 to 5 (yr)	495(19)	377 (30)	0.53 (0.45–0.62)	<0.001***
6 to 17 (yr)	512(19)	116 (9)	2.34 (1.88–2.92)	<0.001***
≥18 (yr)	1539(58)	625 (50)	1.38 (1.21–1.59)	<0.001***
Male	1395 (53)	676 (54)	0.94(0.82–1.08)	0.39
Fever (≥37.8°C)	38 (1)	47 (4)	0.37 (0.24–0.59)	<0.001***
Diarrhea for >4 days	99 (4)	62 (5)	0.74 (0.53–1.04)	0.07
Watery stool	2617 (99)	1205 (97)	3.11 (1.90–5.12)	<0.001***
Bloody stool	10 (>1)	11 (1)	0.43 (0.17–1.08)	0.04*
Abdominal pain	1508 (57)	750 (60)	0.88 (0.76–1.01)	0.07
Vomiting >10 times prior to admission	520 (20)	134 (11)	2.03 (1.65–2.50)	<0.001***
Severe dehydration on presentation	1848 (70)	475 (38)	3.76 (3.26–4.35)	<0.001***
ORS before arrival	2419 (91)	1158 (93)	0.82 (0.63–1.07)	0.14
I.V. before arrival	133 (5)	33 (3)	1.95 (1.30–2.93)	<0.001***
Medication before arrival	1237 (47)	603 (48)	0.94 (0.82–1.08)	0.36
Intravenous rehydration	1714 (65)	468 (37)	3.06 (2.66–3.53)	<0.001***
Monthly income ≤10,000 taka	2242 (85)	981 (79)	1.51 (1.26–1.80)	<0.001***
Treatment of drinking water	1589 (60)	835 (67)	0.74 (0.64–0.86)	<0.001***

Confidence intervals (CI) and *P* values were derived from χ^2^ (chi-square) tests.

*denotes statistically significance.

### Toxin types and colonization factors expressed by ETEC isolated during the study period

Of the isolated ETEC strains, the enterotoxin type were almost equally distributed; ST accounted for 31% of strains; LT/ST for 38% and LT for 31%. The isolation was carried out more or less equally throughout the study period and no seasonality in ETEC enterotoxin type was detected.

In terms of colonization factors (CFs), 47% were positive among the 13 CFs that were tested for in this study. The major CFs detected were CS5+CS6 (15%), CS7 (13%), CS14 (13%), CS17 (13%), CS6 (13%), and CFA/I (9%). The prevalence of CF positive ETECs based on the toxin phenotypes was also determined. When analyzing CFs by toxin type, 35% of LT-producing, 24% of ST-producing and 41% of LT/ST producing ETEC strains were positive for the CFs. The CS7 and CS17 types were expressed mostly by LT expressing ETEC and only about 1% and 3% were LT/ST-ETEC respectively (Table 3).

**Table 3:** Expression pattern of colonization factors for ETEC toxin type (March 2008 and February 2010).

Colonization factors (CFs)	CFs positive ETEC isolatesN = 584(47% of total ETEC )	CFs positive LT Producing ETEC isolatesN = 203	CFs positive ST Producing ETEC isolatesN = 142	CFs positive LT/ST Producing ETEC isolatesN = 239
CS5+CS6	86 (15)	3	11	72
CS7	76 (13)	74	0	2
CS14	75 (13)	8	4	63
CS17	74 (13)	66	0	8
CS6	74 (13)	22	40	12
CS21*	70 (12)	7	33	30
CFA/1	55 (9)	3	30	22
CS4+CS6	26 (5)	3	13	10
CS2+CS3	22 (4)	8	4	10
CS4	10 (2)	0	3	7
CS12	7 (1)	4	1	2
CS8	4 (>1)	3	0	1
CS1+CS3	3 (>1)	1	2	0
CS3	2 (>1)	1	1	0

*With or without other CFs.

## Discussion

Although mortality rates due to diarrhea have decreased globally over the years, diarrheal diseases still range among the highest causes of child and adult morbidity in developing countries in Asia and Africa [25], [26]. The aim of this study was to carry out surveillance for severe diarrhea especially cholera in Dhaka city, which is facing problems of rapid urbanization and the associated lack of public health intervention, sanitation and safe water availability to meet the needs of the growing population [27]. As a result, rates of infectious diseases including cholera have increased tremendously [28], [29].

The equal numbers of male and female patients that sought care from Mirpur were predominantly from lower socioeconomic groups, which reflect the demographic picture that has been seen for diarrheal patients being treated at the ICDDR, B hospital over recent years [30]. The patients who came from the area lacked appropriate water and sanitation facilities in their homes. Almost all patients obtained tap water from the government source. However tap water was collected by household members and then stored in homes for use for drinking, bathing and other purposes [7]. Although 60% reported of having used boiled drinking water, all other usage was from untreated tap water suggesting high risk from such sources. In an earlier study in Mirpur, only 21% of people were found to treat their drinking water and most people also used sanitary latrines [7]. During the study period, more adults than children from Mirpur area attended the hospital, although data from the ICCDR, B 2% surveillance system has shown that 60% of total patients treated are children [31]. The discrepancy could be due to the fact that there are numerous health clinics in and around the Mirpur area, which may cater to the treatment of children when compared with treatment facilities that are available for adults [32], [33].

In terms of causal bacterial pathogens for watery diarrhea in Mirpur patients, *V. cholerae* O1 was the most prevalent 70% in adults with 72% of patients suffering from severe dehydration. However, about 34% of children less than five years diagnosed with cholera suffered from severe diarrhea and required IV fluid requirement; when considering children below the age of 2 years, 12% showed symptoms of severe dehydration. Earlier epidemiological studies in cholera patients have indicated that the disease is more prevalent in older children and adults, which is supported by these study data. However, findings from the Mirpur patients reveal that children are also susceptible to the disease and suffer from severe dehydration [34], [35], [36]. In a 2008 study in Bangladesh, 86% of children 12 years and younger in age that had cholera suffered from severe dehydration [37] while another study has reported cholera in neonates [34]. These reports and the present analyses show the changing trend in cholera epidemiology, which mirrors data published recently in other studies in India and Africa [35], [36], [38].

The biannual seasonality observed for cholera in Mirpur is similar to that seen in most other areas in Bangladesh [3], [5], [30]. Such a typical seasonality suggests that the rising temperature from spring onwards, as well as other environmental factors such as lowering levels of surface water and the increased spread of the pathogen in the community by the fecal oral route may be the major causes of the biannual epidemics in the urban area of the city. Therefore, high prevalence of cholera is likely due to the increased bacterial load in the water, as well as the high transmission rates among the population. In a previous study involving cholera patients and their household contacts, the rates of cholera infection were high [39], [40] with two contacts in a household being infected from a hospitalized index case on average [40]. Since the Mirpur area lacks access to pond and river water, the contribution of these systems to increased cholera outbreaks in Mirpur as has been postulated earlier for rural areas in Bangladesh, cannot be extrapolated to the urban area [41].

A reason for the high rates of cholera in children in Mirpur, despite local clinics offering health care to children in the area, may be due to the immunocompromised status of the children. A relatively high proportion of children studied were stunted (HAZ-<2Z score; 48%) or moderately (54%) to severely malnourished (44%). Earlier studies carried out in Mirpur showed that by the age of two years 38% of children were stunted while 58% were underweight [7]. An important limiting micronutrient in children in developing countries is zinc [42]. It is known that over 50% of children are zinc deficient in Bangladesh [43], as well as in the Mirpur area [44], in addition to varying rates of other malnutrition indices and micronutrients [45].

During the surveillance study, identification of etiological agents other than *V. cholerae* O1 was carried out with emphasis on ETEC, the other major bacterial cause of acute diarrhea. Overall, among the two most common bacterial etiologies, *V. cholerae* O1 infections were the most prevalent and was about the twice the rate seen for ETEC diarrhea in the Mirpur area. The overall trend at the ICDDR,B hospital data was also comparable with that of Mirpur (*V. cholerae* O1: 23%; ETEC: 11%). The rates of ETEC diarrhea seems to have undergone a decrease compared to what has been described earlier although it is still the second most common cause of bacterial diarrhea [2], [5], [30], [46]. ETEC diarrhea was seen in around equal proportions of adults and children. Earlier analyses have shown ETEC to be more prevalent in children than adults [9], [46]. The three enterotoxin types of ETEC were isolated in similar proportions in strains obtained from the study patients. Data from the ICDDR, B hospital over the last decade has shown that the prevalence of the different ETEC toxin phenotypes have undergone a change. In studies carried out between 1996–1998, the ST toxin type was more prevalent. In studies carried out more recently in 2007, the LT phenotype predominated [2]. In the present analyses, all the three phenotypes were evenly distributed among the patients. The phenotypic changes in ETEC have been monitored using the similar methods and techniques over the years and therefore not due to changes in assay procedures. Rotavirus was not prospectively tested in specimens obtained from Mirpur but a 2% systematic surveillance data from the ICDDR, B showed that it is also very high among children from the Mirpur area (42%) [47].

Studies on ETEC have shown that the ST, ST/LT phenotype of ETEC are predominantly CF positive while the LT only strains are not [7], [22]. In contrast, the current data shows that the CF expressing strains belonged to the LT phenotype alone in relatively high proportion of ETEC, an observation that supports results from our study in 2007 [2] but different from our analyses earlier in 2000 where LT-ETEC were generally more negative for CFs [9]. In terms of the CFs detected on strains, the major types were CS7 and CS17, as well as CS5+CS6 and CS6-ETECs. The CS1, CS2, CS3 phenotypes were low in prevalence. Therefore, it appears that ETEC diarrheal agents are undergoing changes in both toxin and CF profiles and ETEC vaccine development should take into consideration the changing phenotypes that are being seen.

The prospective analyses of hospitalized patients from Mirpur in urban Dhaka, shows that *V. cholerae* O1 is the major bacterial pathogen and a cause of severe cholera disease. Strategies for the implementation and use of available cholera vaccines are needed in the area to decrease the annual burden of disease. Mirpur represents a socioeconomic group that best reflects the major areas of high cholera burden in the country .Vaccines that can target such high risk groups in the country and in other similar regions will hopefully be able to reduce the disease morbidity and the transmission of pathogens that impact on the life and health of people.

## Supporting Information

Checklist S1 STROBE checklist
(0.08 MB DOC)
